# The Saturday Fund and the Insurance Bill

**Published:** 1911-08-12

**Authors:** 


### THE SATURDAY FUND AND THE INSURANCE BILL.

### SIR SAVILE CROSSLEY'S SPEECH.

At the recent deputation of the British Hospitals Association and other bodies to Mr. Lloyd George, reported in The Hospital of July 29, page 44, Sir Savile Crossley, on behalf of the Hospital Saturday Fund of London, expatiated upon the Fund's position in the light of the Insurance Bill.

The Hospital Saturday Fund, he said, had 350,000 subscribers, and some eight thousand collectors; in all, the payments made through the Fund last year amounted to £42,000. It was managed by a Board of Delegates, which was thoroughly democratic in character, and the working expenses were only 7 per cent., in consequence of a large number of honorary helpers, and in spite of the number of small amounts received. The Board was convinced that the voluntary contributions from employers and employed to hospitals and kindred institutions would be reduced when the National Insurance scheme came into force; that the voluntary system above all must be maintained; that if the State received compulsory contributions from employers and employed, it should also remunerate voluntary hospitals and kindred institutions for the relief these granted to insured persons for whom the State would be liable; that these institutions should receive the actual cost of the expenditure they incurred in granting relief to insured persons, under conditions which would preserve the independence of the voluntary charitable institutions concerned; that the work done by this and similar Funds should be made known to the approved Societies, and to the Health Committees proposed to be established by the Bill. The experience of this Fund with regard to hospital work in the Metropolis it was felt should be placed at the disposal of the Chancellor of the Exchequer.

